# PCR-based generation of shRNA libraries from cDNAs

**DOI:** 10.1186/1472-6750-6-28

**Published:** 2006-06-21

**Authors:** Cheng Du, Baosheng Ge, Zhongfeng Liu, Kai Fu, Wing C Chan, Timothy W McKeithan

**Affiliations:** 1 Department of Pathology and Microbiology, University of Nebraska Medical Center, Omaha, NE 68198-0766, USA; 2 Department of Internal Medicine, University of Nebraska Medical Center, Omaha, NE 68198-7680, USA

## Abstract

**Background:**

The use of small interfering RNAs (siRNAs) to silence target gene expression has greatly facilitated mammalian genetic analysis by generating loss-of-function mutants. In recent years, high-throughput, genome-wide screening of siRNA libraries has emerged as a viable approach. Two different methods have been used to generate short hairpin RNA (shRNA) libraries; one is to use chemically synthesized oligonucleotides, and the other is to convert complementary DNAs (cDNAs) into shRNA cassettes enzymatically. The high cost of chemical synthesis and the low efficiency of the enzymatic approach have hampered the widespread use of screening with shRNA libraries.

**Results:**

We report here an improved method for constructing genome-wide shRNA libraries enzymatically. The method includes steps of cDNA fragmentation and endonuclease MmeI digestion to generate 19-bp fragments, capping the 19-bp cDNA fragments with a hairpin oligonucleotide, and amplification of the hairpin structures by PCR. The PCR step converts hairpins into double-stranded DNAs that contain head-to-head cDNA fragments that can be cloned into a vector downstream of a Pol III promoter.

**Conclusion:**

This method can readily be used to generate shRNA libraries from a small amount of mRNA and thus can be used to create cell- or tissue-specific libraries.

## Background

RNA interference (RNAi) provides a powerful tool for silencing gene expression. Large-scale phenotypic or pathway-driven screens of siRNA libraries may help to identify novel genes that may be targets for therapy in cancer and other diseases. Two different methods have been used to construct genome-wide siRNA libraries. One is to chemically synthesize oligonucleotides based on siRNA design algorithms (for reviews, see [1,2]). Typically, the oligonucleotides are synthesized in the form of double-stranded DNA molecules containing short hairpin RNA (shRNA) templates and are cloned into a Pol III-driven expression vector. Libraries constructed with this method and targeting more than 10,000 different human genes have been successfully used for screening [3,4]. The other method is to convert collections of cDNAs into shRNA templates. Three groups have developed protocols to produce genome-wide shRNA libraries [5-7]. These protocols share several common features, and all "measure" the appropriate length of the hairpin using the type IIS restriction endonuclease MmeI, which cuts 20/18 nt from its recognition site. The common steps, with minor variations, include (1) generating random cDNA fragments; (2) ligating the cDNA fragments with a double-stranded oligonucleotide that contains an MmeI site; (3) restriction digestion with MmeI; (4) ligating a second oligonucleotide to the digested cDNA fragments to form a double-stranded DNA with a hairpin structure; (5) using a DNA polymerase with strong strand-displacement activity to convert the hairpin DNA into double-stranded DNA; and (6) cloning the double-stranded DNA into an expression vector.

The chemical synthesis method is a very expensive and time-consuming approach that requires synthesis of thousands of oligonucleotides, followed by cloning and sequence validation. Construction of shRNA libraries from cDNAs provides an economical alternative. However, the multiple-step process in the current protocols makes the overall efficiency low and thus requires a large amount of starting mRNA. To increase the efficiency of library construction, we have developed an improved method, which includes newly designed oligonucleotides and a key PCR step to amplify and convert the hairpin structures in the abovementioned step 5 into double-stranded DNAs. The PCR amplification of the hairpin structures greatly increases the overall efficiency of the procedure and allows libraries to be constructed from small amounts mRNA.

## Results and discussion

### The "YIU" procedure

**Figure 1:**
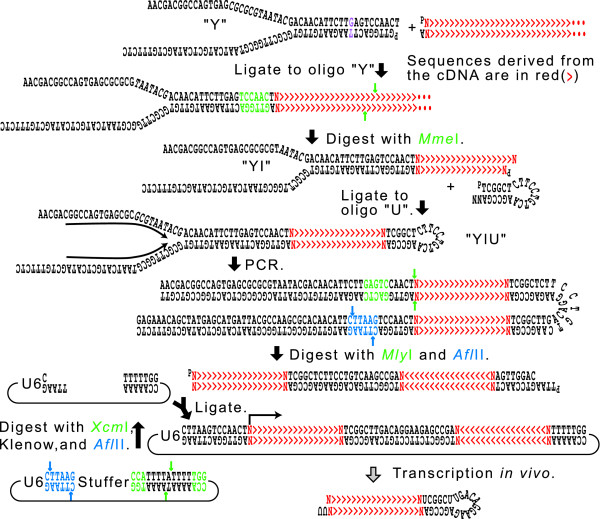
See text for details. A single G-T mismatched is shown in purple. Restriction enzyme recognition sites are shown in green or blue, and sites of cleavage by arrows. The structure of the KSU6 vector is shown in the lower left.

**Figure 2:**
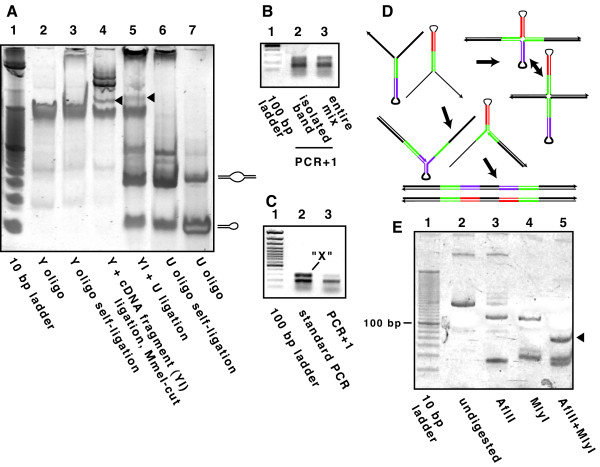
**Generation of shRNA constructs using YIU.** (A) Intermediate products produced during the YIU procedure. Lanes 2 and 3. The Y oligonucleotide, designed with a single 3' T overhang and therefore unable to self-ligate. Lane 2, without ligase. Lane 3, with ligase. Note that the forked shape of the Y oligonucleotide causes abnormal mobility on PAGE. The ligated and MmeI-digested YI molecules are indicated by an arrowhead in lane 4; the digested YI molecules shift in migration when ligated to the U oligonucleotide (arrowhead, lane 5). Note that the YIU molecules are a minor fraction of the product molecules because the Y and U oligonucleotides were added at a large molar excess over cDNAs and that the ligation was efficient, as almost all of the U oligonucleotides have been converted to dimers. (B) PCR amplification of YIU ligation products. The template used for PCR in lane 2 was the PAGE-purified DNA band corresponding to the one labeled with arrowhead in lane 5 of Panel A. The template for lane 3 was the whole YIU ligation mixture shown in lane 5 of Panel A. The calculated size of YIU double-stranded DNA is 160 bp. (C) Conversion of the X molecule into double-stranded DNA by a "PCR+1" protocol. PCR amplification of YIU with 20 cycles results in an extra band (the X molecule) seen in the 200–300 bp region on agarose gels (lane 2). However, when the PCR products are diluted with fresh PCR reaction mix (1 × buffer, dNTPs, primers and enzyme) and subjected to one additional PCR cycle, the high molecular weight band is lost (lane 3). (D) Scheme of the generation of X molecules. Vent polymerase, through its strand-displacement activity, can open hairpin structures to generate fully double-stranded products. As the PCR progresses, however, YIU double-stranded DNA molecules accumulate and the concentration of primers decreases. When the templates are heat denatured and cooled, two heterogeneous molecules (shown in red or purple) can form an X-shaped (cruciform) heterodimer via the common complementary regions (shown in green). (E) Restriction digestion of double-stranded DNA of YIU. The YIU double-stranded DNA generated by PCR (the "uncut" lane) is digested with AflII or MlyI individually, or in combination to generate suitable inserts for cloning.

Fig. 1 outlines our method for converting double-stranded cDNA into a pool of double-stranded DNA comprising a large and diverse population of 19-bp inverted repeats. The process contains three major steps:

(1) Generation of cDNA hairpins with noncomplementary ends. In previous reports [5-7], the first oligonucleotide used either had a CG overhang or was blunt-ended, allowing self-ligation. To avoid this problem, the "Y" oligonucleotide was designed with a single 3'-T overhang (Fig. 1), and thus cannot self-ligate (compare lanes 1 and 2, Fig. 2A). Additional features of the Y oligonucleotide include (a) an embedded MmeI site, (b) long noncomplementary arms designed for anchoring PCR primers with high melting temperatures (see below), and (c) a single basepair mismatch within the 18-bp stem region, resulting in the AflII site and MlyI site each being present on only one arm of the double-stranded PCR products, allowing the products to be directionally cloned. The unusual shape of the Y oligonucleotide causes abnormal mobility on PAGE gels (Fig. 2A). Double-stranded cDNA fragments (the "I") were generated either by restriction enzyme digestion or by partial DNase I digestion and repair with T4 polymerase. They were then treated with *Taq *DNA polymerase to add a single A at the 3' end [8]; this untemplated addition is reported to occur with an average efficiency of around 70% [9]. This treatment prevents self-ligation of cDNA fragments but allows them to ligate to the Y oligonucleotide. Excess "Y" oligonucleotide was added to increase ligation efficiency.

The ligated "YI" molecule was digested with the restriction endonuclease MmeI, a Type IIS restriction endonuclease that cuts 20 and 18 nt away from its recognition site, yielding a 2-nt 3' overhang. A hairpin loop oligonucleotide ("U") with a random 2-nt 3' extension was ligated to the ends generated by MmeI (Fig. 1). The final product ("YIU") contains a 10-nt loop. In "YIU" molecules, the cDNA insert is 19 bp in size. Although the U molecules can ligate to themselves (compare lanes 6 and 7, Fig. 2A), the U dimers cannot be amplified by PCR.

Because the expected product and several intermediates migrate anomalously on PAGE gels, the "YIU" ligation product was identified by excising individual DNA bands from a PAGE gel and PCR-amplifying extracted DNA. One band (arrowhead in lane 5, Fig. 2A), presumably containing the YIU products, gave the maximum yield of the expected 160-bp PCR product (lane 2, Fig. 2B). Alternatively, the entire ligation mix can be used without purification as a template to amplify the 160-bp product (lane 3, Fig. 2B). Thus, it is not necessary to gel-purify the YIU ligation product before PCR.

(2) PCR amplification of "YIU". Regular DNA hairpins (consisting only of a stem and loop) cannot be efficiently PCR-amplified because after the denaturation step, hairpins rapidly re-form upon cooling, precluding primer annealing [10]. However, a DNA hairpin with noncomplementary arms, such as YIU molecules, can be efficiently PCR-amplified [11]. Primers corresponding to the two noncomplementary arms were designed with high melting temperatures, allowing the annealing and extension temperature during PCR cycling to be set at 75°C to destabilize intramolecular hairpins. Vent DNA polymerase was used because of its high fidelity and its strand-displacement activity when at high temperature. Although hairpins presumably form before primer annealing, Vent polymerase can open them by strand displacement (Fig. 2D).

In late cycles of PCR amplification, after the denaturation step, product reannealing competes with primer annealing. In the case of YIU amplification, after denaturation, the single-stranded products will form an intramolecular hairpin. Annealing of two hairpin-containing molecules with complementary arms yields an "X"-shaped (cruciform) product that contains a Holliday junction. Because in a complex library the two strands comprising the X almost always will contain different 19-bp inverted repeats, spontaneous branch migration within the "X" molecules cannot resolve the Holliday junction to generate linear molecules (Fig. 2D). At late PCR cycles, the amplified products consist of a mixture of linear double-stranded DNA duplexes that migrate at the expected 160-bp position and these aberrant structures, which migrate at the 200–300 bp position in agarose gels (lane 1, Fig. 2C, the "X" molecule) and form a smear in PAGE gels (data not shown). To convert the X molecules to linear double-stranded DNA, fresh PCR reaction mix (buffer, dNTPs, primers and polymerase) was added to the PCR product tube, and an additional PCR cycle was performed to synthesize the complementary strand for each strand of the X molecule (lane 2, Fig. 2C). To minimize generation of the X molecules, the optimal PCR cycle number was determined by running four PCR reactions with different cycle numbers. The PCR products were analyzed on agarose gel. With the starting materials used in our experiments, the optimal number of PCR cycles is usually between 16–18. This cycle number was used in scaling up the reactions.

(3) Cloning. The PCR product of YIU was directly digested with MlyI and AflII in the PCR buffer and separated by PAGE (Fig. 2E). The 70 bp product band was excised from gel, purified and directionally cloned into the pKSU6 expression vector.

### Effect of modifications of the U6 promoter and hairpin loop on RNAi

**Figure 3:**
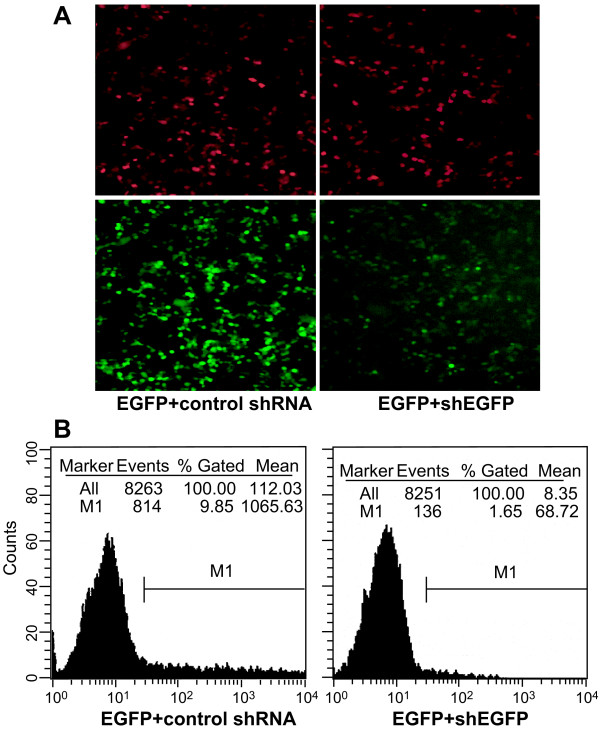
A. Suppression of EGFP expression by a "YIU" shRNA construct in mouse NIH3T3 cells. This shRNA cassette targets EGFP nt 243–261, GCACGACTTCTTCAAGTCC, selected as an effective shRNA cassette by a screen described in the text. When cotransfected with pEGFP vector at a molar ratio of 3 to 1 (shRNA:pEGFP), GFP fluorescence was suppressed; DsRed2-C1 was used as an internal control. Images were taken 24 h after transfection. B. Quantitative analysis of suppression efficiency by flow cytometry. The shRNA expression vector and the GFP reporter vector pMIG, at a molar ratio of 3 to 1, were cotransfected into mouse NIH3T3 cells. A "classic" shRNA expression construct targeting luciferase was similarly cotransfected in control cells. Assays were performed in triplicate at 48 h after transfection. The gate M1 was established using untransfected cells. The suppression, as measured by the decrease in mean fluorescence of the gated population, was quantified by flow cytometry in triplicate for each sample. Note that this is likely to provide a minimal measure of suppression, as GFP expression of many transfected cells may no longer fall within the gate; in fact, the gated population is markedly decreased in cells cotransfected with the shRNA construct targeting EGFP.

The shRNA expression vector pKSU6 contains a modified human U6 promoter region into which an AflII site was introduced for ease of cloning (Fig. 1). To test whether our modifications alter the effectiveness of the resulting shRNAs, a small shRNA library was made from EGFP using DNase I partial digestion and the YIU procedure. Twenty random clones were tested for shRNA efficiency on 3T3 cells when cotransfected individually with the pEGFP plasmid and a control plasmid, pDsRed2, expressing Red Fluorescent Protein (RFP). The fluorescence was examined 24 h after transfection. One construct showing strong suppression by fluorescence microscopy (Fig 3A) was further analyzed by flow cytometry; in these experiments, GFP was driven by the LTR promoter of a retroviral vector, pMIG (MSCV-IRES-GFP). Fig. 3B shows that this shRNA construct could effectively suppress GFP expression at a 3:1 molar ratio; suppression was an average of 96% in four individual assays. Therefore, the modifications appear to adversely affect neither transcription from the U6 promoter nor subsequent shRNA processing.

**Figure 4:**
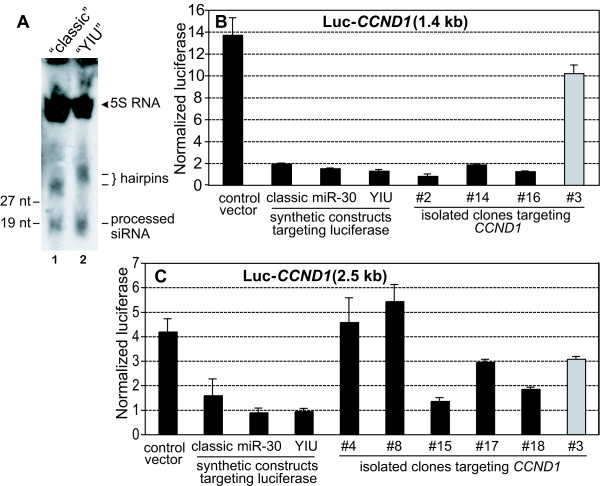
**A**. Total RNA isolated from HEK293T cells transfected with either a "classic" or "YIU" construct targeting luciferase was analyzed by Northern blot hybridization to a radioactively labeled luciferase 19-nt sense-strand oligonucleotide and to a 19-nt oligonucleotide complementary to 5S RNA. 19- and 27-nt single-stranded DNA oligonucleotides were used as size standards; because DNA rather than RNA oligonucleotides were used, the indicated sizes are only approximate. The expected approximate sizes of the hairpin transcripts are 49 nt ("classic") and 62 nt ("YIU"), whereas both processed forms are expected to be ~21 nt. **B**. and **C**. Constructs expressing luciferase mRNA fused with a fragment of *CCND1 *mRNA were cotransfected with shRNA expression constructs targeting either luciferase or *CCND1*, as described in Table 1. The negative control shRNA expression construct was a "classic" construct targeting *BCL2*. Samples were analyzed in duplicate, and values were normalized using *Renilla *luciferase expression. Error bars represent the standard deviation. The shRNA constructs targeting *CCND1 *was cotransfected with a luciferase-*CCND1 *fusion construct containing the target sequence, with the exception of #3, whose target sequence is absent from both fusion constructs. Construct #3 thus serves as an additional negative control. The small degree of suppression seen with #3 and the apparent small stimulation by some shRNA cassettes may be either due to experimental error or to off-target effects of #3 and of the control shRNA construct. The data shown are representative of three individual experiments, which all showed similar results.

To confirm that our modifications do not interfere with transcription or processing, we prepared three constructs targeting the same sequence in the luciferase gene (GTGCGCTGCTGGTGCCAAC), which is known to be an effective target [7]. The "classic" construct uses a U6 promoter, differing minimally from the U6+1 construct of Paul et al. [12], and the loop TTCAAGAGA, corresponding to the earliest generation of shRNA expression cassettes [13]. The "YIU" construct has the structure generated by the YIU procedure. In the "miR-30" construct, the targeting sequence is engrafted into part of the human microRNA-30a primary transcript sequence, contained in the pSHAG-MAGIC2 (pSM2) vector [14]. Fig. 4A shows the results of Northern blot hybridization analyzing cells transiently transfected with equal amounts of the "classic" or "YIU" construct targeting luciferase. The two constructs generated similar quantities of the primary hairpin transcript and the processed siRNA. Functional comparison of the three constructs, as described below, demonstrates that the "YIU" construct is at least as effective as the other two constructs in targeting luciferase.

### Cloning efficiency and quality of the shRNA library

The model system we chose was the human *CCND1 *gene, encoding cyclin D1, which is frequently upregulated in cancer. This upregulation can occur through gene amplification in breast cancer and through a chromosomal translocation in mantle cell lymphoma and other B-cell malignancies. We generated a *CCND1 *shRNA library and analyzed random clones for effective RNAi. The full-length cDNA (4.2 kb) was digested by AluI, DpnI, and HaeIII, all of which are restriction endonucleases that yield blunt ends. Digestion with the three restriction endonucleases generates 54 fragments. Because both ends of the DNAs can be used for ligation, there are 108 possible ligation products in total. After cloning into pKSU6, random bacterial colonies were picked, and PCR was performed to examine the inserts. Out of 95 colonies, 71 (75%) had inserts. Twenty clones were sequenced (Table 1). Eighteen of the twenty clones have hairpin inserts; one clone yielded only the *lacZ *gene sequence, implying that recombination had occurred; and one clone contains a longer *CCND1 *fragment. In the 18 clones with hairpins, the inserts are either 19 or 20 bp, reflecting the variability in the site of cleavage by the MmeI endonuclease [5]. All the 18 clones derived from a restriction digestion: 8 clones from HaeIII, which has the most sites (28 sites) in *CCND1*, 5 clones from AluI (17 sites), 4 clones from DpnI (9 sites), and 1 clone from EcoRI (1 site). The positions of the inserted sequences were random within *CCND1*: 5 clones were from nt 1–1000; 6 from nt 1001–2000; 3 from nt 2001–3000; and 4 from nt 3001–4200. Importantly, the PCR step of the YIU process appears to amplify all hairpin structures with no apparent bias with respect to GC content. In the 18 sequenced clones, the percentage of GC varied between 30% and 84%, with 5 clones (28%) having less than 50% GC, 6 clones (33%) having 50–60% GC, and 7 clones (39%) having GC content over 60%. Therefore, our two-temperature PCR cycle protocol can amplify a wide range of potential hairpin structures.

### Analysis of the CCND1 shRNA library

To demonstrate the utility of this shRNA library construction method, random clones from the *CCND1 *shRNA library were tested for effective RNAi by cotransfection with one of two constructs expressing luciferase fused in its 3'UTR to a portion of the *CCND1 *gene (Fig. 4B and 4C). The shRNA constructs were also compared to three constructs targeting the luciferase portion of the fusion transcript. The two *CCND1 *fragments together comprise almost the entire *CCND1 *cDNA with the exception of a small portion of the 3' UTR containing a cluster of AUUUA motifs, which are often associated with mRNA instability [15]. The relatively low level of expression of the "2.5 kb" construct suggests, however, that this fragment may confer instability of the fusion transcript; this may result in less apparent further destabilization by the shRNA expression constructs. A destabilizing region has been mapped within the sequences contained in the "2.5 kb" construct [16,17]. Notably, the YIU construct targeting luciferase was at least as effective as either the "classic" construct or the construct in which the target is embedded in a microRNA structure ("miR-30"). Several of the enzymatically generated constructs effectively targeted the fusion transcript. The most effective of the tested clones, #2, is predicted to be a potentially effective shRNA by Dharmacon's program [18].

Construction of shRNA libraries is the first step toward the goal of performing mammalian genome-wide screens with the RNAi technology. The applications of shRNA library technology also include determining the best sequences for inhibition of infection by viruses, such as HIV [19,20], and identifying the most effective shRNAs for a single gene.

Given the great amount of work required for a functional screen, if the construction of an shRNA library becomes much easier, tissue- or cell-specific shRNA libraries are preferable to generic synthetic shRNA libraries for the following reasons: (a) Not all genes have been identified. This is particularly likely to be the case for genes with a very limited range of tissue expression. Thus, with our current state of knowledge, any library prepared by individual chemical synthesis is necessarily incomplete. (b) Alternative splice forms of some genes may not be affected if only one or a few shRNAs are used. (c) Screening only for genes that are actually expressed in a given tissue reduces the amount of work required. Normalized, tissue-specific cDNA libraries from IMAGE collections may be the most cost-effective source of genome-wide cDNA libraries for generating shRNA libraries. However, each laboratory can pursue its specific interests using appropriate tissue-specific shRNA libraries.

A major step in constructing an shRNA library from cDNA is to normalize the cDNA. A simple cDNA normalization method has recently been reported [21]. The key component in this method is a duplex-specific nuclease (DSN) from the Kamchakta crab. The DSN preferentially cleaves double-stranded DNA and DNA in RNA-DNA hybrid duplexes at 70°C. Using this enzyme, cDNA can be normalized after first-strand cDNA synthesis or after amplifying the cDNA. After heat-denaturation and kinetic reassociation [22,23], abundant cDNAs reanneal more rapidly than rare cDNAs and are depleted from the mixture by DSN digestion.

## Conclusion

The YIU method allows rapid conversion of cDNAs into shRNA templates. This method has several advantages over previous methods [5-7]. Our Y oligonucleotide was designed with a 3' T overhang to prevent self-ligation. To allow ligation with the Y oligonucleotide, a single 3' A overhang is added to cDNA fragments by incubating with *Taq *DNA polymerase. To create multiple fragments from each transcript, cDNAs derived from cells or tissues can be subjected to either partial DNase I digestion, sonication, or restriction digestion. A PCR step after generating the YIU molecules was introduced to amplify the desired product but not irrelevant byproducts, thereby eliminating the necessity of multiple PAGE purifications as described in previous methods [5-7] and greatly simplifying the whole process. The PCR amplification also increases the overall yield of YIU products and allows the use of small amounts of starting mRNA. This is expected to be particularly useful for small number of cells separated by FACS or microdissection.

The two-temperature PCR program used a high temperature (75°C) for a combined annealing and extension step to destabilize hairpin structures and to promote the strand-displacement activity of Vent polymerase. This approach has been used in amplifying DNA with a hairpin structure [11], and we showed that this method has additional applications in the siRNA field, such as amplification of shRNA expression cassettes. Conventional 3-temperature PCR cycles promote strand slippage of DNA polymerase when replicating self-annealing structures [24,25], including the shRNA hairpin [14], leading to deletions. Adoption of our PCR program to the application may alleviate the problem.

Finally, the YIU method is versatile. If an initial larger loop is secondarily trimmed using a Type IIS restriction enzyme, constructs can be produced expressing an shRNA with a loop of arbitrary sequence [5-7]. In particular, with minor changes in the Y and U oligonucleotides, one potentially could prepare shRNA expression libraries in which the targeting siRNA sequences are engrafted into a microRNA stem and loop structure. The use of a microRNA stem and loop has been shown to dramatically increase the efficiency of suppression by an shRNA [14] and is compatible with Pol II promoters, which are more diverse and versatile than the Pol III promoters necessary for expression of "classic" shRNAs.

## Methods

### Oligonucleotides

The "Y" oligonucleotide pair: Y-Forward: 5'-AACGACGGCCAGTGAGCGCGCGTAATACGACAACATTCTTGAGTCCAAT-3'. Y-Reverse: 5'-PO_4-_GTTGGACTTAAGAATGTTGTGCGCTTGGCGTAATCATGGTCATAGCTGTTTC-3'. The "U" oligonucleotide: 5'-PO_4_-TCGGCTCTTCCTGTCAAGCCGANN-3'.

PCR primers. Forward: 5'-CGACGCCCAGTGAGCGCGCGTAATACG-3'. Reverse: 5'-GGAAACAGCTATGACCATGATTACGCCAAGCG-3'.

### cDNA fragmentation

Human *CCND1*, encoding cyclin D1, from the 4.2 kb full-length cDNA clone MGC:23386 [Genbank:BC023620] (American Type Culture Collection), used here as an example, was isolated from vector sequences with EcoRI and XhoI (yielding 2.7 and 1.5 kb fragments, due to an internal EcoRI site) and end-blunted with Klenow fragment. The gel-purified *CCND1 *fragments (about 1 to 1.5 μg) were digested in 1 × ThermoPol buffer (New England Biolabs, NEB) at 37°C for 1 hour individually with AluI, DpnI and HaeIII, restriction endonucleases with a 4-bp recognition sequence and blunt-end products. Concentrated dNTPs (final 200 μM each) and *Taq *polymerase (1 U) were added into each tube and incubated at 60°C for 3 hours. After combining the three samples, DNA was extracted with phenol/chloroform, precipitated by ethanol, and resuspended in 10 μl of H_2_O (about 0.3 μg/μl).

The EGFP gene was PCR-amplified from plasmid pEGFP-C2 (BD Clontech) using *Taq *DNA polymerase (NEB). After PCR, 1 M Tris-HCl, pH 7.0, and 0.1 M MnCl_2 _were added to final concentrations of 40 mM and 1 mM, respectively. Partial digestion with DNase I (0.1 U in 50 μl) was performed at room temperature for several time periods (2 to 10 minutes) to determine the optimal digestion time. The partially digested DNA samples were extracted with phenol/chloroform and precipitated with ethanol. DNA end-repair with T4 DNA polymerase (NEB) was conducted following the manufacturer's protocol. The addition of a 3' A overhang was described above.

### The "YIU" procedure

**(1) **"YI" ligation. Three μl of digested DNA (about 0.9 μg, the "I" molecule, shown in red in Fig. 1) was mixed with 1 μl of 10 × NEB buffer 4, 1 μl of 0.1 M DTT, 1 μl of 10 mM rATP, 3 μl of 10 μM "Y" paired oligonucleotide and 1 μl T4 DNA ligase (2,000 NEB units, New England Biolabs) and placed in a thermocycler overnight, cycling between 10°C for 30 sec and 30°C for 30 sec [26]. The T4 DNA ligase was inactivated at 65°C for 20 min.

**(2) **MmeI digestion. 1.5 μl of 1 mM S-adenosylmethionine (SAM),1 μl MmeI (2 U, NEB), 1 μl of 10 × NEB buffer 4, and 6.5 μl of water were added to the YI ligation mix (final 20 μl in volume), and incubated at 37°C for 1 hour.

**(3) **YI and "U" ligation. The MmeI-digested DNA was extracted with phenol/chloroform, precipitated with ethanol, and resuspended in 30 μl of water. Four μl of 10 × T4 DNA ligase buffer, 5 μl of "U" oligonucleotide (10 μM), and 1 μl T4 ligase (400 NEB Units) were added, and the mixture incubated in a thermocycler overnight, cycling between 10°C for 30 sec and 30°C for 30 sec.

**(4) **PCR amplification. To determine the optimal number of PCR cycles, four tubes were prepared, each containing 1 μl of the YIU ligation product, 1 unit of Vent DNA polymerase (NEB) and 1 × buffer, 200 μM dNTPs and 0.5 μM of primers (final conc.) in 50 μl of volume. PCR was carried out with the following cycling parameters: 95°C for 2 min, 95°C for 30 sec, 75°C for 1 min, for 15, 17, 19, or 21 cycles. After PCR amplification, the products were separated by 1.5% agarose gel electrophoresis. The band patterns from the four samples were compared, and the cycle number yielding the greatest quantity of the 160 bp product was chosen for amplifying a larger quantity for cloning. After the PCR, an equal volume of fresh PCR reaction mix (buffer, dNTPs, primers and polymerase) was added, and one additional cycle was performed.

**(5) **Cloning. Restriction endonucleases AflII and MlyI were added directly to the PCR product and incubated at 37°C for 2 hours. The 70 bp insert was separated on a 12% polyacrylamide-TBE gel, purified, and cloned into the expression vector pKSU6.

### The shRNA expression vector pKSU6

The U6 promoter was PCR amplified from vector pAVU6+27 [12] with primers 5'-GGAAGATCTGAGGAGGGCCTATTTCCCATG-3' (forward) and 5'-CCGGAATTCCTTAAGTTCCACAAGATATATAACTCTATC-3' (reverse). The PCR product was digested with BglII and EcoRI and inserted into the BamHI-EcoRI sites in pBluescript II KS+ (Stratagene). An XcmI site was introduced downstream of the AflII site. The Pol III transcription termination signal (T_5_) was embedded in the XcmI site (CCAAAAATTTTTTGG). The resulting vector, pKSU6, was cut with XcmI, blunt-ended with T4 DNA polymerase, and subsequently cut with AflII for cloning AflII-MlyI digested YIU products. The ligated products were transformed into *E. coli *XL-10 competent cells (Stratagene).

### Control constructs targeting luciferase

The "YIU" construct targeting luciferase was generated by preparing the KSU6 vector as described above and cloning into it a double-stranded oligonucleotide prepared by annealing the oligonucleotide 5'-ttaaGTCCAACTGTGCGCTGCTGGTGCCAACTCGGCTTGACAGGAAGAGCCGAGTTGGCACCAGCAGCGCAC-3' and its complement (without ttaa). The U6 promoter of pBtU6+27 [12] was amplified using the following primers: 5'-CCGCGGTACCCCGGGAGATCCAAGGTCGGGCAG-3' (forward) and 5'-CGCGTCTAGACCCATCGATGAGGATCCCTTTCCACAAGATATATAAAGCC-3' (reverse). The PCR product was digested with KpnI and XbaI and cloned into the corresponding sites of pBtU6+27. The resulting construct, pU6-ClaI, is similar to pKSU6, but contains BamHI and ClaI cloning sites at the 3' end of the U6 promoter. The oligonucleotide 5'-cgTGCGCTGCTGGTGCCAACTTCAAGAGAGTTGGCACCAGCAGCGCACTTTTT-3' and its complement (with 5' ctag, and lacking 3' cg) were annealed and cloned into the ClaI and XbaI sites to yield the "classic" construct targeting luciferase. The "miR-30" construct was prepared by PCR amplification using primers 5'-CAGAGGCTCGAGAAGGTATATTGCTGTTGACAGTGAGCG-3' and 5'-CTAAAGTAGCCCCTTGAATTCCGAGGCAGTAGGCA-3' and template 5'-TGCTGTTGACAGTGAGCGAGGTTGGCACCAGCAGCGCACTTAGTGAAGCCACAGATGTAAGTGCGTTGTTGGTGTCAATCCTGCCTACTGCCTCGGA-3', essentially as described [14]. The product was digested with XhoI and EcoRI and cloned into the corresponding sites of the pSM2 vector (Open Biosystems).

### Cyclin D1 (CCND1) reporter vector preparation

pGL3 Promoter construct (Invitrogen) was modified by inserting into the XbaI site a double-stranded oligonucleotide prepared by annealing the following two oligonucleotides: 5'-ctagGAATTCGATATCCCGCGGCATATGT-3' (forward) and 5'-ctagACATATGCCGCGGGATATCGAATTC-3'. A construct with the insert in the forward orientation was denoted pGL3P(2MCS), having a second multiple cloning site within the 3'UTR of luciferase. Fragments of *CCND1 *were inserted into this second multiple cloning site. Construct luc-*CCND1*(2.5 kb) was prepared in several steps, including deletion of a BstXI fragment, removing approximately 1.6 kb of 3' sequence; it includes nt 1–2173 [Genbank:BC023620] of *CCND1 *and additional 3' sequences. Construct luc-*CCND1*(1.4 kb) was prepared by cloning an EcoRI-XbaI fragment of *CCND1 *(nt 2750–4197) into the corresponding sites of pGL3P(2MCS). These two reporter constructs were chosen to avoid a cluster of AUUUA sequences, which in some mRNAs are associated with mRNA instability [15].

### Cell culture and transfection

Human HEK293T or mouse NIH3T3 cells were used for RNAi assays. The cells were seeded 24 hours before transfection in DMEM plus 10% FCS in 96-well plates at 50–70% confluence. Transfection was conducted with Lipofectamine 2000 (Invitrogen) according to the manufacturer's recommendations. The amounts of DNA used per well were as follows: the reporter plasmid, pEGFP, 50 ng; Red Fluorescent Protein vector (pDsRed2, Clontech), 50 ng; and shRNA expression vector, 100 ng (~3 × molar ratio).

### Northern blot hybridization

Hybridization was performed as previously described [27], with minor modifications. HEK293T cells were transiently transfected with either the "classic" or "YIU" shRNA construct targeting luciferase; total RNA was extracted with Trizol reagent. 40 μg of total RNA was electrophoresed on a 15% polyacrylamide gene in TBE buffer (89 mM Tris-borate, 2 mM EDTA) with 8 M urea. RNA was transferred to GeneScreen Plus membrane (PerkinElmer) with a semidry electrophoretic transfer apparatus and UV-crosslinking with 1000 μJ in a Stratagene UV Crosslinker. StarFire™ template probes (Integrated DNA Technologies, IDT) contained the following sequences: targeting luciferase, 5'-GTGCGCTGCTGGTGCCAAC-3', and 5S RNA, 5'-GACGAGATCGGGCGCGTTC-3'. Probes were labeled with [α-^32^P]-dATP using the Nucleic Acids Labeling System kit (IDT), following the manufacturer's protocol. The membrane was prehybridized in 0.5 M sodium phosphate buffer, pH 7.0, 1 mM EDTA, 7% SDS, 0.5% (w/v) sodium pyrophosphate at 37°C. Approximately 1.7 × 10^6 ^dpm of the luciferase probe and 4 × 10^5 ^dpm of the 5S RNA probe were added to 2 ml of hybridization buffer. After hybridization at 37°C for ~15 h, the blot was washed 3 × with 2 × SSPE (2 × buffer, 0.3 M NaCl, 20 mM sodium phosphate, pH 7.4, 2 mM EDTA), 0.1% SDS 30 min each at 37°C and then exposed overnight to X-ray film.

### Luciferase assays

HEK293 cells in 24-well plates were cotransfected with 300 ng Luc-*CCND1 *reporter plasmid, 900 ng shRNA expression plasmid, and 20 ng pRL-TK (*Renilla *luciferase control). Samples were extracted 48 h after transfection and analyzed using the Dual-Luciferase Reporter Assay System (Promega), according to the manufacturer's protocol.

## Authors' contributions

CD carried out the YIU procedure, constructed the *CCND*1 shRNA library, participated in the design of this study, conducted most of the experiments, and drafted the manuscript. BG carried out vector construction. ZL generated some of the constructs and carried out the luciferase assays. KF and WCC participated in the design and helped to coordinate the study and to draft the manuscript. TWM conceived and coordinated this study, developed the overall strategies, and helped to draft the manuscript. All authors read and approved the final manuscript.

## Figures and Tables

**Table 1:** *CCND1*-YIU library.

No.	Position	Restriction Site	Sequence	Size (bp)	%GC	Orient-ation
1	3727–3746	AluI	CTCACAGTGCTGTGTGCCCC	20	65	+
2	3497–3516	AluI	CTATGGAAGTTGCATAATTA	20	30	+
3	2455–2473	HaeIII	GCAGAGGATGTTCATAAGG	19	47	-
4	1692–1711	HaeIII	CCACGCTACGCTACTGTAAC	20	55	-
5	1934–1952	HaeIII	CTGGTTTTCTACCCAACGG	19	53	+
6	846–864	DpnI	TCAAGTGTGACCCAGACTG	19	53	+
7	765–783	HaeIII	CCGCAGTGCAAGGCCTGAA	19	58	+
8	594–613	HaeIII	CCGCAATGACCCCGCACGAT	20	60	+
9	3318–3336	DpnI	TCCCACACAGGCTGGCGGG	19	74	+
10	312–331	DpnI	TCGTCGCCACCTGGATGCTG	20	65	+
11	1057–1075	HaeIII	CCGGCTCCGCCCTCGCTGC	19	84	-
12	3424–3442	DpnI	TCAGATGAAGTGCCCAGCA	19	53	-
13	1804–1822	HaeIII	GTAGCAGGGTCGGGAAAGG	19	63	+
14	2970–2988	AluI	CTCCATTTTCTTATTGCGC	19	42	+
15	1989–2007	AluI	CTACTTGGTTTGTGTTCTT	19	37	+
16	2752–2771	EcoRI	AGGGGCAGGGGATAAGAATT	20	50	-
17	359–377	HaeIII	CCAGCGGGAAGACCTCCTC	19	68	-
18	1117–1135	AluI	TTGATACCAGAAGGGAAAG	19	42	-
19	Recombined		*LacZ*			
20	Long *CCND1*			62		

*CCND1 *sense-strand sequences are shown. Nucleotide numbers correspond to [Genbank:BC023620]. Orientation is denoted "+" or "-" if the sequence shown occurs before or after the shRNA loop, respectively.
